# Deep learning architectures for multi-label classification of intelligent health risk prediction

**DOI:** 10.1186/s12859-017-1898-z

**Published:** 2017-12-28

**Authors:** Andrew Maxwell, Runzhi Li, Bei Yang, Heng Weng, Aihua Ou, Huixiao Hong, Zhaoxian Zhou, Ping Gong, Chaoyang Zhang

**Affiliations:** 1 School of Computing, University of Southern Mississippi, Hattiesburg, MS 39406 USA; 2 Cooperative Innovation Center of Internet Healthcare, School of Information & Engineering, Zhengzhou University, Zhengzhou, 450000 China; 3 Department of Big Medical Data, Health Construction Administration Center, The Second Affiliated Hospital of Guangzhou University of Chinese Medicine, Guangzhou, China; 4 Division of Bioinformatics and Biostatistics, National Center for Toxicological Research, US Food and Drug Administration (FDA), Jefferson, AR 72079 USA; 5 Environmental Lab, US Army Engineer Research and Development Center, Vicksburg, MS 39180 USA

**Keywords:** Deep neural networks, Deep learning, Intelligent health risk prediction, Multi-label classification, Medical health records

## Abstract

**Background:**

Multi-label classification of data remains to be a challenging problem. Because of the complexity of the data, it is sometimes difficult to infer information about classes that are not mutually exclusive. For medical data, patients could have symptoms of multiple different diseases at the same time and it is important to develop tools that help to identify problems early. Intelligent health risk prediction models built with deep learning architectures offer a powerful tool for physicians to identify patterns in patient data that indicate risks associated with certain types of chronic diseases.

**Results:**

Physical examination records of 110,300 anonymous patients were used to predict diabetes, hypertension, fatty liver, a combination of these three chronic diseases, and the absence of disease (8 classes in total). The dataset was split into training (90%) and testing (10%) sub-datasets. Ten-fold cross validation was used to evaluate prediction accuracy with metrics such as precision, recall, and *F*-score. Deep Learning (DL) architectures were compared with standard and state-of-the-art multi-label classification methods. Preliminary results suggest that Deep Neural Networks (DNN), a DL architecture, when applied to multi-label classification of chronic diseases, produced accuracy that was comparable to that of common methods such as Support Vector Machines. We have implemented DNNs to handle both problem transformation and algorithm adaption type multi-label methods and compare both to see which is preferable.

**Conclusions:**

Deep Learning architectures have the potential of inferring more information about the patterns of physical examination data than common classification methods. The advanced techniques of Deep Learning can be used to identify the significance of different features from physical examination data as well as to learn the contributions of each feature that impact a patient’s risk for chronic diseases. However, accurate prediction of chronic disease risks remains a challenging problem that warrants further studies.

## Background

Chronic diseases are responsible for the majority of healthcare costs worldwide [1, 2]. An early diagnosis from an expert can help save a patient in terms of healthcare costs and extend the lifespan and quality of life for a patient. Early diagnosis of a chronic disease is often difficult due to the complexity and variability of the factors that lead to the disease. In an effort to help physicians diagnose these types of diseases early, computational models are being utilized to predict if a patient shows signs of one or more types of chronic diseases. The advantage of modern big data analysis allows physicians to infer information from patient data with less computational time and cost. This will allow physicians to build powerful tools for the purposes of intelligent health risk prediction.

Recently, deep learning techniques are being used for all different purposes with great success and are becoming more popular within various disciplines. Because of its generality, similar architectures put together through deep learning can be applied to many classification problems. Particularly within the medical field they are increasingly being used as a tool for multi-label classification. For example, Mayr et al. use a Deep Neural Network as a way to identify different sets of chemical compounds for toxicity prediction for humans [3], Lipton et al. use Recurrent Neural Networks to analyze time-series clinical data to classify 128 different diagnoses [4], and Esteva et al. use Convolutional Neural Networks to identify skin-cancer [5].

In this study, hypertension, diabetes, and fatty liver are three chronic diseases that are analyzed to predict types of chronic diseases for a patient. The diagnosis that is given for a certain patient can be one of the three, some combination of the diseases, or can be diagnosed as showing no signs of any of the diseases. This means that overall there are eight different diagnoses that can be given.

The layout of the paper is as follows: Methods will describe the two Deep Learning architectures that were used as a predictor for the multi-label classification dataset, the different types of algorithms that serve as a benchmark for comparison purposes, and explain evaluation methods that show how Deep Learning architectures perform when compared against traditional and other similar multi-label classification type methods; Results will describe the data and report the differences of performance between the methods chosen; Finally, discussion and conclusions are made about the performance of deep learning architectures for the purposes of predicting chronic diseases in physical examination records.

## Methods

Several different machine learning methods are brought together to compare the performance of Deep Learning architectures on the physical examination data. In this section, combinations of traditional machine learning methods are used, plus there are a few methods that were specifically developed to solve multi-label classification problems. The other traditional methods can be used to solve multi-label problems, but generally involves some manipulation of the dataset in order for the algorithm to interpret targets of a dataset correctly. In other words, it transforms a multi-label dataset into a single-label dataset with multiple classes. There are many different techniques that have been used to handle this type of conversion. There are generally two categories for multi-label classification problems: problem transformation or algorithm adaption methods. One of the more popular problem transformation techniques is called the Label Powerset (LP) [6], where each unique set of labels for a multi-label dataset is considered a single label. This unique set of labels is considered a powerset. A classifier is trained on these powersets in order to make a prediction. Some of the following methods make use of this particular technique in order to handle multi-label classification. However, there are some drawbacks when manipulating the data to suit this format. It is common for LP datasets to end up with a large amount of represented classes and few samples of each class to train on. An advantage that Deep Learning methods have over similar problem transformation techniques is that it can train on the original data without needing to resort to some type of conversion of the data. These Deep Learning methods fall more into the algorithm adaptation category.

### Ensemble methods

There are a couple of methods that were used to compare against the Deep Learning techniques that make use of, or have a variation of, the LP transformation. In particular, the Random *k*-Labelsets (RAkEL) method for multi-label classification [7, 8] is one such method that utilizes LPs to train on groups of smaller, randomly selected sets of labels, which are of size *k*, using different classifiers on groups of LPs, then uses a majority voting rule as the basis for selecting target values. If the average of the predictions for a label is above a certain threshold, then the label is chosen as true for that instance.

The ELPPJD method [9] is an ensemble multi-label classification method that uses a technique similar to LP and RAkEL where the data is transformed into a multi-class problem, then performs a joint decomposition subset classifier method to handle imbalanced data. This joint decomposition creates subsets of the data based upon the number of samples per LPs.

### Classifiers

The following section describes the classification methods that we used for prediction. Besides the Deep Learning methods, most of these classifiers were part of a single label, multiclass step when used with RAkEL and MLPJTC after the dataset transformation. These classifiers were the “base” classifiers for the previous mentioned multi-label classification methods.

A Multilayer Perceptron (MLP) [10] is a machine learning method that was originally developed to try and discover if researchers can simulate how a brain operates. As researchers added more improvements to this method such as backpropagation [11], it became one of the more common classification tools because of the way that the network could infer information about the data in the absence of a priori information. The architecture of an MLP is usually described as having a network of layers where there are at least three layers: an input layer, hidden layer, and an output layer. Each of these layers is built with multiple different nodes that have edges, or weights, connecting to each successive layer in the network. Each node in the network calculates the synaptic weight of the connections of the previous layer and then passes the results of this to an activation function, usually some sigmoidal type of function. Eq. 1 shows the calculation of the synaptic weight of a single node at position *j* and all previous *N* edges connected to the node with some additional bias *b*, which is generally random Gaussian noise defined as *b*~*N*(0, 1). In Eq. 1, *X*_*i*_ is the input node of the previous layer node position (*i*) with feature length *N* in the network and *W*_*ij*_ is the associated weight for the link connecting node *i* in the previous layer and the node *O*_*j*_ in the current layer. Eq. 2 represents the activation function of the node, where *ϕ* is the sigmoid function, but could easily be any number of other activation functions such as the hyperbolic tangent function.

1 $$ {O}_j=\sum \limits_{i=1}^N{X}_i{W}_{ij}+{b}_j $$

2 $$ \phi =\frac{1}{1+{e}^{-\left({O}_j\right)}} $$

The number of nodes for an input layer is typically the features or attributes of a dataset, and the connections of the input layer to the hidden layer can be different depending on how many nodes are selected for the hidden layer. The hidden layer can consist of multiple different layers stacked together, but it is generally assumed that the performance of an MLP does not increase past two layers. The hidden layer is connected to the output layer, where the output layer is the same number of classes that are getting predicted. The calculation above happens for each node in the network until the output layer is reached. At this point, called a forward pass, the network has tried to learn about the sample passed in, and has made a prediction about that data, where the nodes of the output layer are probabilities that the sample is of a certain class. This is at the point where backpropagation takes over. Since this is a supervised technique, an error between the prediction *y*_*j*_ and the target *t*_*j*_ of the sample *n* is calculated as the difference between the two values (Eq. 3) and passed to a loss function (Eq. 4) to determine a gradient, which allows the network to adjust, or back propagate, all of the weights between each node up or down depending upon the gradient of the error (Eq. 5). Eq. 5 shows the equation for a gradient descent method. In general, it is an optimization function min_*θ*_(*ε*(*n*| *θ*)) where *θ* is the vector of parameter values. Δ*w*_*j*_(*n*) represents the change in weight for the node at position *j* for sample *n*, *α* is a parameter called the learning rate, which determines how much to move in the direction of the gradient, *y*_*i*_ is the prediction from the output layer, and $\frac{d}{dn}\varepsilon \left(n|\theta \right)$ is the gradient of the loss function.

3 $$ {e}_j={t}_j(n)-{y}_j(n) $$

4 $$ \varepsilon \left(n|\theta \right)=\frac{1}{N}\sum \limits_j^N{e}_j^2(n) $$

5 $$ \Delta {\mathrm{w}}_{\mathrm{j}}\left(\mathrm{n}\right)=-\upalpha \frac{\mathrm{d}}{\mathrm{d}\mathrm{n}}\upvarepsilon \left(\mathrm{n}|\uptheta \right){\mathrm{y}}_{\mathrm{i}}\left(\mathrm{n}\right) $$

This process of a forward pass and backpropagation continues until a certain number of iterations are met, or the network converges on an answer. Another way to look at the method is that the architecture is using the data to find a mathematical model or function to best describe the data. As the network is trying to learn, it is constantly searching for a global minimum value such that predictions can be accurate.

The C4.5 algorithm [12] is a classification method that is used to build a decision tree. It uses the concept of information gain and attributes of the data to split nodes of a tree into one class or another. It decides the best attribute of the data to properly split samples of the data and follows some base cases to add more nodes to the tree.

Support Vector Machines (SVM) work by trying to separate the classes from samples of a data into different hyperplanes. It tries to maximize the distance between classes as much as possible. It can use one hyperplane for linear classification, or it can have an infinite number of hyperplanes for nonlinear classification. The way that this is achieved is utilizing kernel functions that have the ability to linearly separate the data.

For this study, there were two different implementations of SVM algorithms that were tested with the physical examination dataset. One implementation used Sequential Minimal Optimization (SMO) [13] while the other is a slight variation of the SMO algorithm that was developed from the library package LibSVM [14, 15].

Random Forest is another decision tree type algorithm that takes advantage of the concept of bagging, or using many different learned models together to make an accurate prediction [16]. It creates a collection of different decision trees based on random subsets of samples per tree and decides which class to predict by employing a voting mechanism to rank the decisions.

ML-KNN is an extension of the *k* nearest neighbors algorithm for multi-label classification [17]. It works by determining the *k* nearest neighbors for an instance as it is passed to the algorithm, then the information gained from the labels that are determined to be mostly associated with the instance is used to predict the appropriate LP for the unseen instance. BP-MLL is multi-label neural networks algorithm that can be considered for performance comparison, which will be included in our future work. This algorithm was successfully applied to classification of functional genomics and text categorization [18].

### Deep learning architectures

Deep Learning architectures are becoming more popular as a set of tools for machine learning. For multi-label classification, these types of systems are performing very well, even sometimes outperforming humans in certain aspects. Here, Deep Learning methods are used to predict chronic diseases for intelligent health risk prediction. What follows is a brief description of the types of architectures that we implemented when using physical examination records to predict chronic diseases. There are two different implementations of the DNN used for multi-label classification: one for problem transformation, and another for algorithm adaptation.

Deep Neural Networks (DNN) are an extension of the MLP and is usually considered a DNN if the MLP has multiple hidden layers [19, 20]. In addition to multiple layers, there are different types of activation functions and gradient descent optimizers that help to achieve a solution to an issue that MLPs suffer from which is the vanishing gradient problem. The vanishing gradient problem arises whenever a network is trying to learn a model, but the gradients of an error are so small that adjustments to the weights through backpropagation almost make no difference to the learning process and gets to a point of never reaching a global minimum. As mentioned before, there are different activation functions that are typically used for MLPs and DNNs, such as sigmoid or hyperbolic tangent functions. However, specifically for Deep Learning, different activation functions have been proven to achieve better results in certain cases. One of these activation functions is called a Rectified Linear Unit (ReLU). For some activation functions, the evaluation of a node can lay between negative one and positive one. However, for the ReLU function, an evaluation that is below zero is cut off and the value can only be between zero and one, or more formally *f*(*x*) = max(0, *x*) where *x* is the result of the equation coming from the node of the network. Gradient descent optimizers are optimization algorithms used for the purposes of finding a local minimum. Hyper parameters such as learning rates and momentum serve these gradient descent algorithms by shifting how much to move through a function space in order to converge on a global minimum. If a value is either too low or too high then the optimizer may miss the global minimum entirely and focus on a local minimum, or perhaps it may never converge at all.

To optimize the hyper parameters of these deep learning networks we opted to go with a grid search to find the best solution and let the networks converge on a model that suits the data. A grid search is one in which there are multiple different variables one should account for in a deep learning model to reach the global minimum as fast or as accurate as possible. For the multilayer perceptron, there were three different parameters: epochs, learning rate, and hidden layers. In practice, these are the parameters that changed prediction results the most. Epochs are how many iterations of the data the network will be used to train a model, the learning rate is how fast or slow the gradient decent optimizer adjusts to reach the minimum, and hidden layers refer to the number of individual layers between the input and output layers. The DNNs in our example are fully connected networks, meaning that each node contains a connecting edge to all of the nodes in the successive layer in the network. Hidden layer units are the number of nodes that exist in each individual hidden layer in the network. The number of units that were chosen came to be 35. This is based on one of the parameters that WEKA uses for their multi-layer perceptron, where they use the equation *a* = (*attributes* + *classes*)/2 to determine some number of units for a layer.

There are also some different activation functions that were used, either the sigmoid function or ReLU, and dropout layers were also chosen. Dropout was developed for the purposes of helping a network avoid overfitting [21]. The basic idea behind dropout is to block certain nodes from firing in the network and allow other nodes the opportunity to learn through different connections or infer different information by only allowing access to certain information. There are differing opinions on whether or not one should allow dropout between each layer, or only during the last hidden layer and output. In this study both options are investigated to get an overall view of how the network performs.

Determining the cost function for a network can make a large difference in the accuracy of the network so special care should be taken to examine whether or not the right cost function is used. For single label data, a softmax function was used for the output layer. The reason for this is straightforward. The equation for the softmax function is as follows:

6 $$ \sigma {(n)}_i=\frac{e^ni}{\sum_{k=1}^K{e}^nk}\mathrm{for}\ i=1\dots K $$

where a vector of *n* values of length *K* is normalized against the exponential function. The idea behind the softmax function is to normalize the data such that the values of the output layer in the network lie in the range (0, 1) and the sum total of the values equal 1. These values can then be interpreted as probabilities, where the highest probability is most likely the best candidate label for the sample in the dataset. Of course, this is acceptable for single label data because each label is considered mutually exclusive. For multi-label data another option should be considered. Because we cannot use softmax in this case, we should use some other function that has a range of (0, 1) so that these can be interpreted as probabilities. The sigmoid function is a good use for this task. Since the predictions in the output layer of the network are independent of the other output nodes, we can set a threshold to determine the classes for which the sample belongs. In our case, the threshold ***θ*** for the output layer is 0.5 (Eq. 7). When selecting θ, analyzing the output of the prediction values to find the range will help to guide selection of the threshold value.

7 $$ \boldsymbol{f}\left(\boldsymbol{x}\right)=\left\{\begin{array}{c}0,\kern0.5em \boldsymbol{x}<\boldsymbol{\theta} \\ {}1,\kern0.5em \boldsymbol{x}\ge \boldsymbol{\theta} \end{array}\right.,\boldsymbol{where}\ \boldsymbol{\theta} =0.5 $$

### Evaluation methods

In order to compare these different methods, accuracy cannot be the single metric used to determine the effectiveness of an algorithm. There are multiple other methods that typically get used to get an overall census on how a method performs. For example, one method could have a very high accuracy, but the data could be imbalanced and the model could be biased towards some certain class that dominates the dataset and only selects that class as the prediction based on the training data, ensuring that most of the guesses are labeled correct even though it is simply selecting the dominating class most of the time without actually learning any information about the data.

The metrics that are used to compare the different methods are accuracy, precision, recall, and F-score. The accuracy of a method determines how correct the values are predicted. Precision determines the reproducibility of the measurement, or how many of the predictions were correct. Recall shows how many of the correct results were found. F-score uses a combination of precision and recall to calculate a score that can be interpreted as an averaging of both scores. The following equations show how to calculate these values, where *TP*, *TN*, *FP*, and *FN* are true positive, true negative, false positive, and false negative respectively.

$$ Accuracy=\frac{TP+ TN}{TP+ FP+ TN+ FN} $$

$$ Precision=\frac{TP}{TP+ FP} $$

$$ Recall=\frac{TP}{TP+ FN} $$

$$ F\  Score=\frac{2\times Precision\times Recall}{Precision+ Recall} $$

### Classifier evaluation platform and development environment

The majority of classifiers were used with the software package WEKA, which as mentioned earlier is a common benchmark tool to evaluate the performance between multiple algorithms. There are two different categories of classifiers that were used with WEKA; one that used the GUI interface to run individual algorithms on the data that was transformed via the MLPJTC method, and the other category used the MULAN package that was built upon the WEKA API to handle the multi-label data. For multi-label classification, the RAkEL method from the MULAN package is used, and then the base classifier implemented through the WEKA API is used for classification of the data itself. In other words, the RAkEL method transforms the multi-label data in order for the classifiers to be run. The MLPJTC results are listed in Table 1 and the RAkEL results are listed in Table 2. An additional multi-label method, MLkNN, is also listed in Table 2. MLkNN was implemented in the MULAN package by the authors of RAkEL and is a method that was included for benchmark purposes. The deep learning architectures were implemented in the deep learning package TensorFlow, which is an API written in Python and developed by Google. TensorFlow provides a way to build deep neural networks using basic implementations of the different deep learning architectures, or the axioms of these architectures. TensorFlow also includes tools to evaluate performance and help with deciding how to manipulate parameters to allow the network to learn properly.

**Table 1:** The results of the classifiers for single-label, multi-class dataset

Algorithm	Accuracy (%)	Precision	Recall	F-Score
LibSVM	49.89	0.422	0.499	0.416
MLP	74.94	0.744	0.749	0.744
SMO	69.67	0.691	0.697	0.670
J48	77.26	0.771	0.773	0.771
DNN	71.10	0.757	0.711	0.726
RF	81.51	0.810	0.815	0.808

**Table 2:** The results of the classifiers for multi-label dataset

Base Classifier	Accuracy (%)	Precision	Recall	F-Score
RAkEL-LibSVM	59.47	0.697	0.603	0.630
RAkEL-MLP	81.63	0.854	0.838	0.837
RAkEL-SMO	59.47	0.697	0.603	0.630
RAkEL-J48	83.64	0.864	0.865	0.856
RAkEL-RF	85.67	0.884	0.880	0.874
MLkNN	51.03	0.602	0.530	0.547
DNN	92.07	0.915	0.867	0.823

## Results and discussion

### Dataset and preprocessing

The physical examination dataset is from a medical center where 110,300 anonymous medical examination records were obtained [9]. In the table of dataset, each row represents the physical examination record of a patient and each column refers to a physical examination item or feature, except for the last six columns that indicate disease types. The dataset includes 6 normal chronic diseases including hypertension, diabetes, fatty liver, cholecystitis, heart disease, and obesity and the prediction in this study focuses on the first three of them. Each type of six diseases corresponds to a class label in the classification. From over 100 examination items, 62 features were selected as significant based on expert knowledge and related literature. These items are 4 basic physical examination items, 26 blood routine items, 12 urine routine items, and 20 items from liver function tests. One may get more details about the dataset from [9] and website provided at the end of this paper.

In order to get some evaluations on the data, a ten-fold cross validation step is performed on the data, where 90% of the data is used for training and 10% is left for testing. Usually, random sampling is enough to get results from cross validation, however with the physical examination records another approach is needed because not all classes were being represented in the training for the model of the classifier.

**Fig. 1:**
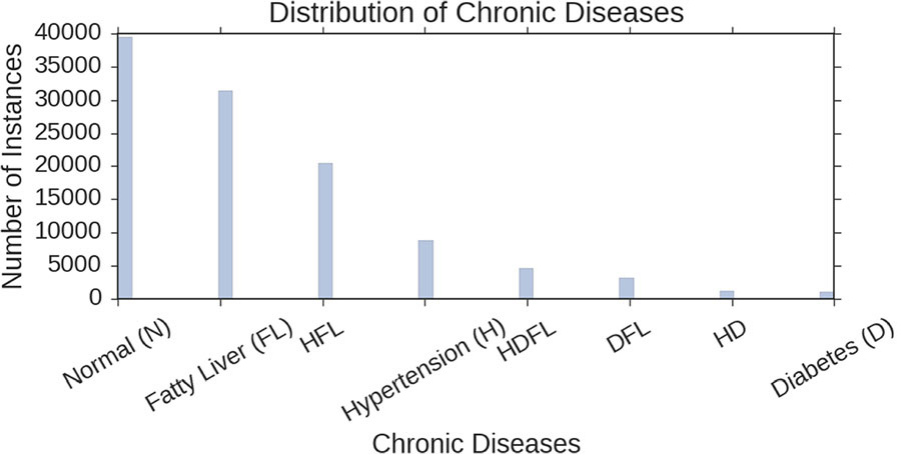
The distribution of physical examination records for chronic diseases. Here, the list of chronic diseases are Fatty Liver (FL), Diabetes (D), Hypertension (H), a combination of these diseases (DFL, HFL, HD, HDFL), and the absence of the disease or classified as Normal (N)

From Fig. 1, when the data is transformed into a single label, multiclass problem it is apparent that there is a vast amount of imbalance in the data. This was a bit expected considering that we were transforming the dataset using the LP method. As mentioned in the beginning of the paper, it is common to end up a situation such as this, where some labels have a small representation of the overall dataset. The first two classes alone make up for 64.25% of the data. With such an imbalanced dataset, it is not hard to imagine that a classifier could tend to be biased towards the first two classes. A couple of strategies were employed to help the classifiers avoid biased predictions. The first is to stratify the training and testing datasets when randomly sampling for a ten-fold cross validation. Stratifying a dataset in this case means that the sampling is proportional to the original dataset. In other words, the sampling will maintain the percentage of class labels from the original data, but will ensure that each class is represented for training purposes. Another issue presented itself however because the lower classes did not have enough samples for the model to differentiate between specific instances when training. A way to help with this is to include oversampling of the lower classes so that more information can be gained for lower represented classes. One such implementation is the Synthetic Minority Over-sampling Technique (SMOTE) [22]. This method under-samples the majority class as well as over-samples the minority classes and additionally introduces some synthetic examples of the minority to fill some feature space for the class rather than simply oversample with replacement or making multiple copies of instances. According to the authors of SMOTE, this is an improvement technique that has worked well with handwritten character recognition.

### Comparison of different classifiers

In Table 1, various popular classification methods are compared against each other to analyze the performance of the single-label, multi-class dataset. LibSVM and SMO are different types of support vector machines, MLP is the WEKA implementation of the Multilayer Perceptron, J48 is the Java implementation of the C4.5 decision tree algorithm, DNN represents the deep learning architecture that was implemented in TensorFlow, and RF is the Random Forest classifier.

The support vector machines were not able to handle the data as well as the decision tree type algorithms, which scored the best overall. MLP and DNN similarly scored lower than the decision tree algorithms. In the case of single label, multi-class, a bagging type algorithm does fairly well on this dataset.

For Table 2, the classifiers from Table 1 are used as a base classifier for the RAkEL method in order to handle multi-label classification. The difference here is in the MLkNN and DNN methods. These two methods could handle the data without first transforming it into a LP. In all cases of RAkEL except for SMO, the results were improved from the previous table. MLkNN performed the worst out of all methods. DNN had the best accuracy, but when considering the other metrics listed in the table, RAkEL with Random Forest as a base classifier was the best performing classifier overall. This makes sense, because not only is RAkEL creating random subsets of the data, but Random Forest is also generating subsets of the samples for its decision trees. This allows for a very large coverage of all the features to be able to strongly identify correlations in the data. These subsets could allow for more precision when making a prediction. The DNN architecture is trying to find correlations from the data as a whole without any type of ranking, voting, or making subsets of the samples, so there is a wider net of interpretation from the dataset. Also, different adjustments of hyper-parameters could help increase precision and recall values. This dataset in particular has a large amount of TN values which dominate the terms in the equation for accuracy. The model itself tended toward a negative prediction. This is one reason why accuracy was so high while other metrics were lower.

### Optimization of deep learning parameters in single-label data

A grid search of hyper parameters was used when trying to find the optimal parameter to use with the physical examination dataset. When using a grid search one could randomly choose a set of parameters and train using the chosen set, then repeat until a certain number of runs were achieved, or another option would be to iterate through all possible combinations to get performance metrics for each run. The latter was chosen as the preferred method of evaluation in addition to the ten-fold cross validation step. The epochs, or iterations were 775 and 1000, the learning rate was 0.01, 0.05, 0.75, and 0.1. Hidden layers for the single label data were set as either 1 or 2. The Sigmoid and ReLU activation functions were also used for comparisons to evaluate how each of them compared.

**Fig. 2:**
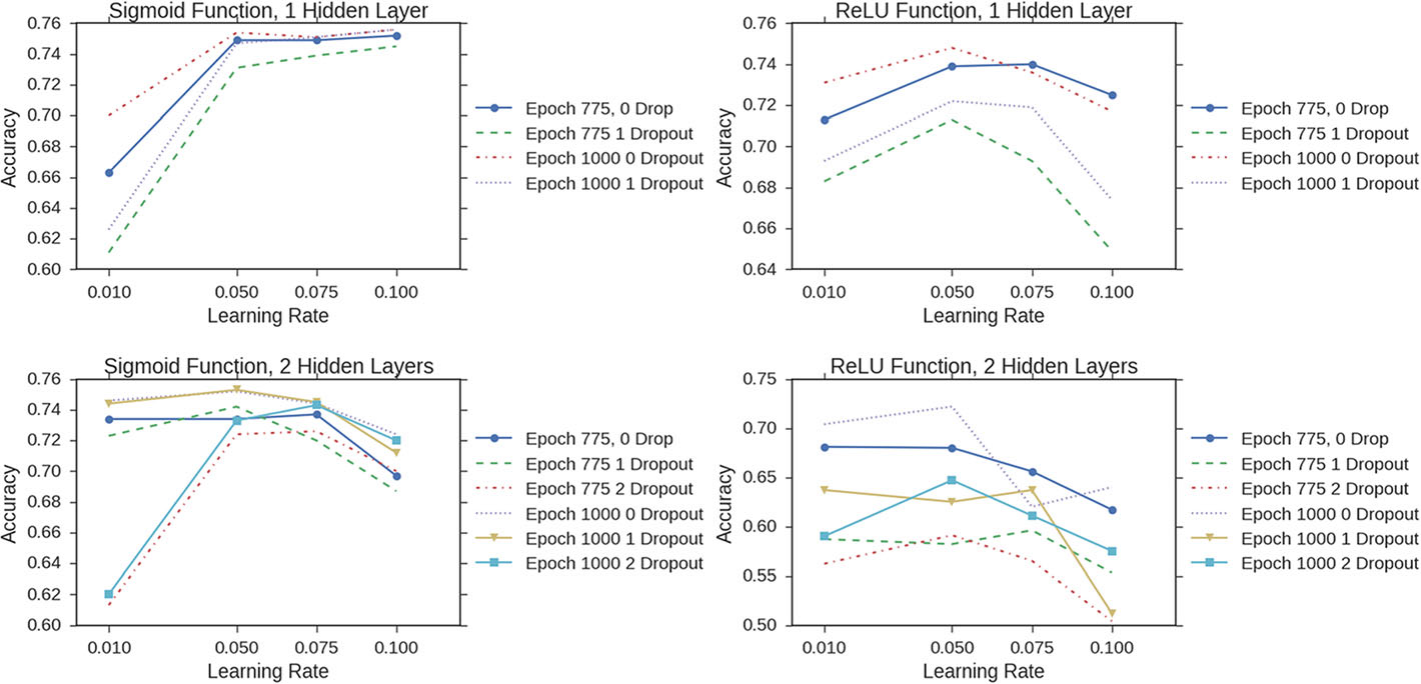
Performance comparison of activation functions. The sigmoid and ReLU activation functions are compared against each other in the DNN architecture

**Fig. 3:**
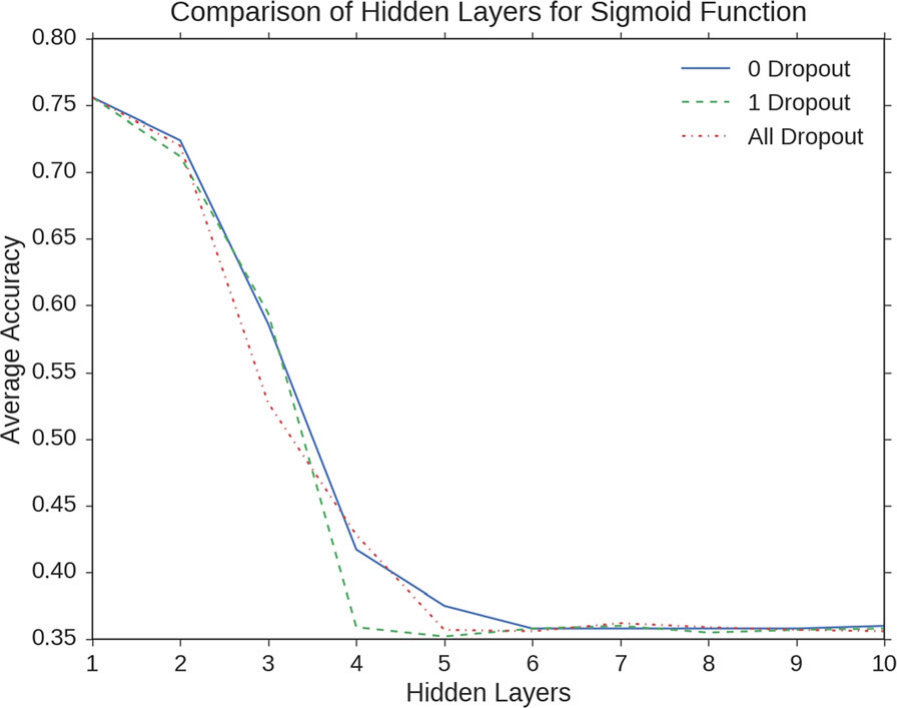
A comparison of additional layers added to the MLP. The hyperparameters are: 1000 epochs, 0.1 learning rate, 35 hidden layer units, hidden layers from 1 to 10, and no dropout to one dropout layer to all dropout layers. These parameters were chosen because they gave the best overall performance for MLP with 1 or 2 layers

Overall, the Sigmoid function performed better than the ReLU activation function when compared with the same hyper parameters, as shown in Fig. 2. Further analysis was performed to see the effects of adding multiple layers had on the network to learn about the data. As you can see from Fig. 3, accuracy drops drastically as multiple layers are introduced. The simpler the network is constructed, the better the accuracy becomes. Here, there are multiple dropout layers introduced to compare performance, including no dropout layers, one dropout layer between the last hidden layer in the network and the output layer, and dropout layers between every layer in the network. The results given in Fig. 3 show that when the network is past the fourth hidden layer, the network plateaus in performance. As more layers are introduced to the network, the issue of the vanishing gradient is more apparent and propagates to the other layers in the network more quickly as a consequence. In addition, for such a problem as this, the extra layers added more complexity to the model that may not reflect the complexity of the data itself.

The structure of the DNN here is very similar to the implementation of the MLP provided by the WEKA software benchmark tool. However, there are some differences which accounts for the variation in the results. In terms of nodes in the network, each represented node in the WEKA version uses the sigmoid activation function including the output layer. For the loss function, the squared-error loss is used with backpropagation for learning. In the case of the TensorFlow implementation, the output layer of the network was made up of linear units that were not squashed by any activation function. For the loss function, a softmax function with cross entropy was used to calculate the error across the network, then it is passed to an optimizer that implements the Adam algorithm [23] for stochastic gradient optimization.

### Impact of deep learning parameters for multi-label data

The following results are using the DNN architecture without any transformation of the data (algorithm adaptation) in order to obtain results for multi-label classification. The architecture is almost identical to the single label, multiclass data, however the cost function has changed. As previously mentioned, the cost function for this architecture has to be a bit different considering the fact that a prediction for a class is not mutually exclusive, so the sigmoid function with the addition of cross entropy was selected and a threshold (***θ***) is used on the results of the cross entropy calculation to determine whether or not a class is predicted for multi-label classification. It was found that the sigmoid activation function performed better than the ReLU and hyperbolic functions for this case. To verify that the results were consistent, different numbers of units per layer were tested. In Table 3, the DNN for multi-label data has the same hyper parameters as the previous best version, but the numbers of units per layer were tested with 35, 256, and 512 units. Similarly, the single label version also had better overall results from a less complex architecture, but because of the LP of the data, the distribution of classes were so varied and imbalanced that the metrics suffered some loss in the results. Particularly in the multi-label data, the accuracy seems to be better than other multi-label methods that were compared. Accuracy does generally give an overall view of the results of the architecture itself, but more importantly the other metrics such as precision, recall, and f-score truly give a better sense of the performance of the network. In the case of the DNN for multi-label data, the training metrics are pretty high, but the metrics for the testing data are lower than the training data. This indicates that the testing data has some wide variability that the network cannot grasp.

**Table 3:** DNN results for multi-label data with respect to different number of units

Units Per Layer	Accuracy (%)	Precision	Recall	F-Score
35	92.07	0.915	0.867	0.823
256	91.34	0.919	0.854	0.798
512	91.80	0.917	0.865	0.819

The specific architectures that were developed for the physical examination data were DNNs. However, there are a variety of different architectures that could have been chosen. In this case, it seemed that other architectures did not perform as well as DNNs, possibly due to the fact that the data itself is not so complex as to need the level of computation that other architectures like Convolutional Neural Networks or Recurrent Neural Networks would need. In addition to the complexity, the learning method of the data generally would fit a regression type of model to learn against the data, which does not necessarily fit the type of data that is generally associated with the other architectures. In most cases, such a type of classification of this data falls in the category of DNNs.

**Fig. 4:**
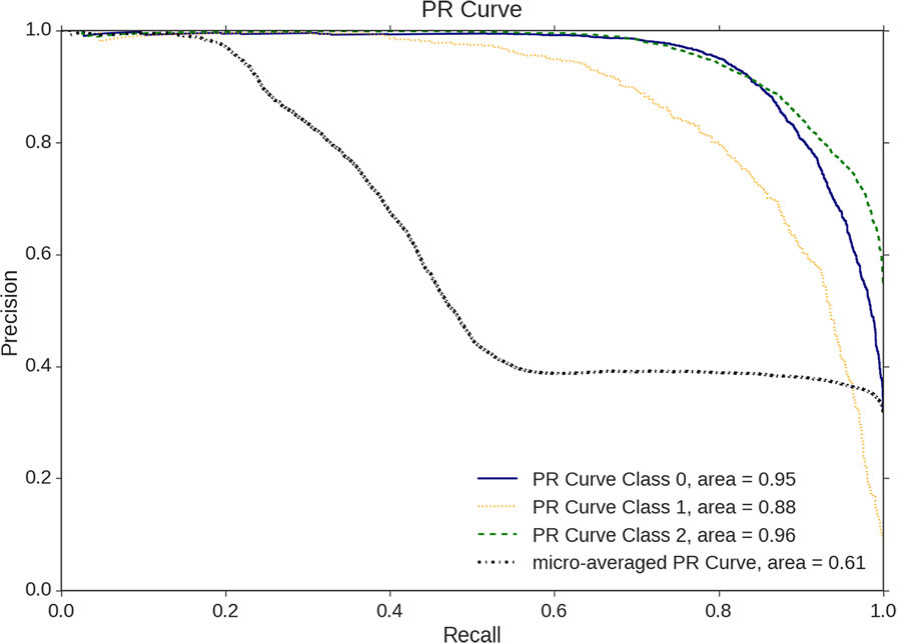
The Precision Recall (PR) curve for the testing dataset. The testing dataset which contained 10% of the data, or 11,030 instances. Class 0 is Hypertension, Class 1 is Diabetes, and Class 2 is Fatty Liver

**Fig. 5:**
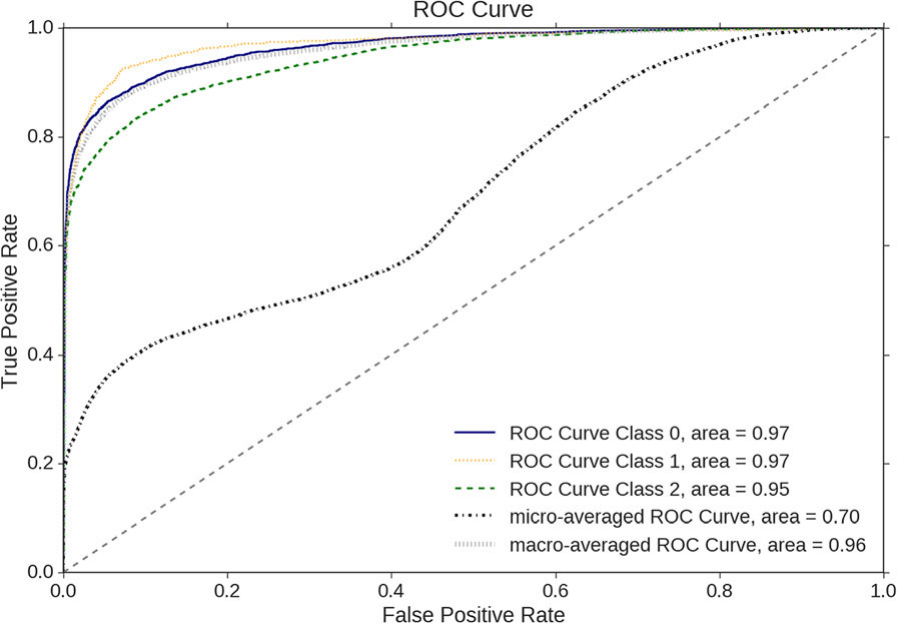
The Receiver Operator Characteristic (ROC) curve of the testing data. Class 0 is Hypertension, Class 1 is Diabetes, and Class 2 is Fatty Liver

Figure 4 and Fig. 5 show the area under the precision-recall curve (AUPR) and the area under the receiver operating characteristic curve (AUROC). These two values combined together show the overall performance of a trained classifier, and have been used many times to determine the effectiveness of a model to predict a class [24]. The performance of the classifier is determined from each class independent of the other, and then together as micro and macro averaged scores. A micro-averaged score gives a value that considers the weight of each class label, whereas the macro-average score is an averaging of the individual scores across each label. The equations for micro and macro scores are shown below.

$$ {Precision}_{micro}=\frac{\sum_{i=1}^l{TP}_i}{\sum_{i=1}^l{TP}_i+{FP}_i}\kern0.48em {Precision}_{macro}=\frac{\sum_{i=1}^l\frac{TP_i}{TP_i+{FP}_i}}{l} $$

$$ {Recall}_{micro}=\frac{\sum_{i=1}^l{TP}_i}{\sum_{i=1}^l{TP}_i+{FN}_i}\kern0.48em {Recall}_{macro}=\frac{\sum_{i=1}^l\frac{TP_i}{TP_i+{FN}_i}}{l} $$

For the multi-label dataset, an increase in accuracy could be explained by the fact that each class has more training samples since the classes are not mutually exclusive. Considering the distribution of each LP in Fig. 1, the imbalanced data is less of an issue and each class is more likely to have some representation when random sampling for the training set. Some adjustment could be made to the threshold value when the prediction of the output layer is calculated, which could also improve the accuracy of the model.

The introduction of batch normalization has also improved the results of the training [25]. Batch normalization is the process in which *mini-batches* of the training data are used to step through the network instead of processing the entire training dataset as one step of training. The reason is to minimize the impact of the covariate shifts from the features of the input data, effectively normalizing the layers and reducing the need for other architecture regularization techniques such as dropout layers. Another advantage is that batch normalization can reduce the amount of epochs needed to train the network. For example, before batch normalization, our network achieved an accuracy of 89.90%, after 1000 epochs. After batch normalization using a batch size of 512, the accuracy increased to 92.07%, with only 100 epochs, significantly reducing the amount of training time.

Some architectures can be sensitive to initialization weights. Although the purpose of a Neural Network is to be able to adjust weights even from random initial values, setting the initial weights can significantly affect the results of the prediction depending on the architecture. In the described implementation, a truncated normal is used to initialize the weights within two standard deviations from the mean. The standard deviation was selected to be 0.001 with a mean of zero, so the random values ranged between 0 and 0.003. Previously implemented architectures used a randomized normal distribution for values ranging between zero and one, but selecting a truncated normal so close to zero increased all evaluation measures by a few points. This architecture seemed to learn fairly well no matter the initialization values. Evaluation measures varied only a small amount.

## Conclusions

In this study, a multi-label classification method is developed using deep learning architectures for the purposes of predicting chronic diseases such as hypertension in patients for physicians. Such architectures are valuable tools as they are able to calculate correlations in the data through iterative optimization techniques. The results show that DNNs give the highest accuracy among all six popular classifiers. The F-score of DNNs is slightly lower (but compatible) than Random Forrest and MLP classifiers and but much higher than that of SVM and MLKNN classifiers. DNNs play a valuable role in the future of multi-label classification methods because they are able to adapt to the original data and can eventually find a decent optimized function even with rudimentary pieces from which to learn information. Some expert knowledge could vastly improve the rate and ease at which a network could learn the intricate details of a system. In this case, there are some areas of improvement that could be made in terms of the architecture and a thorough investigation of the way the data is passed through the architecture of the network should be considered. Further modification of this architecture could enhance the performance of the model in order to achieve better results for precision, recall, and f-score values. Deep learning architectures provide a powerful way to model complex correlations of features together to form an optimized function from which physicians can predict chronic diseases. Additional improvements to the model could easily allow for the inclusion of other chronic diseases as newer data is gathered.
